# Quantitative retinal morphology and mortality in individuals with proliferative diabetic retinopathy: a retrospective cohort study in a large real-world population

**DOI:** 10.1136/bmjopen-2025-105231

**Published:** 2025-09-09

**Authors:** Abdullah Zafar Khan, Ana Paula Ribeiro Reis, Abraham Olvera-Barrios, Yukun Zhou, Dominic J Williamson, Robbert R Struyyen, Hagar Khalid, Catherine Egan, Alastair K Denniston, Pearse A Keane, Siegfried K Wagner

**Affiliations:** 1 University College London, London, UK; 2 University College Dublin School of Medicine, Dublin, Ireland; 3 NIHR Biomedical Research Centre, Moorfields Eye Hospital NHS Foundation Trust, London, UK; 4 NIHR Birmingham Biomedical Research Centre, Birmingham, UK

**Keywords:** Diabetic retinopathy, Medical retina, Health informatics, DIABETES & ENDOCRINOLOGY

## Abstract

**Abstract:**

**Objectives:**

To investigate whether quantitative retinal markers, derived from multimodal retinal imaging, are associated with increased risk of mortality among individuals with proliferative diabetic retinopathy (PDR), the most severe form of diabetic retinopathy.

**Design:**

Longitudinal retrospective cohort analysis.

**Setting:**

This study was nested within the AlzEye cohort, which links longitudinal multimodal retinal imaging data routinely collected from a large tertiary ophthalmic institution in London, UK, with nationally held hospital admissions data across England.

**Participants:**

A total of 675 individuals (1129 eyes) with PDR were included from the AlzEye cohort. Participants were aged ≥40 years (mean age 57.3 years, SD 10.3), and 410 (60.7%) were male.

**Outcome measures:**

The primary outcome was all-cause mortality. Quantitative retinal markers were derived from fundus photographs and optical coherence tomography using AutoMorph and Topcon Advanced Boundary Segmentation, respectively. We used unadjusted and adjusted Cox-proportional hazards models to estimate hazard ratios (HR) for the association between retinal features and time to death.

**Results:**

After adjusting for sociodemographic factors, each 1-SD decrease in arterial fractal dimension (HR: 1.54, 95% CI: 1.18 to 2.04), arterial vessel density (HR: 1.59, 95% CI: 1.15 to 2.17), arterial average width (HR: 1.35, 95% CI: 1.02 to 1.79), central retinal arteriolar equivalent (HR: 1.39, 95% CI: 1.05 to 1.82) and ganglion cell-inner plexiform layer (GC-IPL) thickness (HR: 1.61, 95% CI: 1.03 to 2.50) was associated with increased mortality risk. When also adjusting for hypertension, arterial fractal dimension (HR: 1.45, 95% CI: 1.08 to 1.92), arterial vessel density (HR: 1.47, 95% CI: 1.05 to 2.08) and GC-IPL thickness (HR: 1.56, 95% CI: 1.03 to 2.38) remained significantly associated with mortality.

**Conclusions:**

Several quantitative retinal markers, relating to both microvascular morphology and retinal neural thickness, are associated with increased mortality among individuals with PDR. The role of retinal imaging in identifying those individuals with PDR most at risk of imminent life-threatening sequelae warrants further investigation.

**STRENGTHS AND LIMITATIONS OF THIS STUDY**

- This study leverages a large real-world retrospective cohort of patients with proliferative diabetic retinopathy (PDR).
- Quantitative retinal features were extracted from routinely collected multimodal retinal images using validated automated pipelines.
- Only patients with available imaging after PDR diagnosis were included, leading to the exclusion of some individuals with unavailable or earlier imaging.
- Cause-specific mortality data were not available and potential confounders such as medication history could not be accounted for.

## Introduction

 Diabetes mellitus is a leading cause of premature mortality, disability and health-system costs worldwide.^1^ In 2024, 589 million adults across the world were estimated to have diabetes.^2^ In the UK, as of 2024, more than 5.8 million people are estimated to be living with diabetes.^3^ Diabetic retinopathy is a common and potentially vision-threatening complication of diabetes. In the USA in 2021, it was estimated that 9.60 million people (26% of those with diabetes) had diabetic retinopathy and 1.84 million people (5% of those with diabetes) had vision-threatening diabetic retinopathy.^4^ The association between diabetic retinopathy and mortality has been well described in individuals with non-proliferative diabetic retinopathy (NPDR) having a 1.15–1.40 fold increase in all-cause mortality compared with age-matched controls.^5^,^7^ Proliferative diabetic retinopathy (PDR) is the most advanced form of diabetic retinopathy and is defined by the growth of new and abnormal blood vessels within the retina and/or optic nerve head. As a marker of end-organ damage, PDR is intimately related to morbidity and mortality, with a recent meta-analysis estimating that affected individuals had a 2.3 times increased risk of death.^7^ Within the UK national diabetic retinopathy screening programme, the 10-year survival in patients with PDR was only 76%.^8^ Individuals with PDR have also been found to have the greatest risk of systemic vascular comorbidities such as stroke, coronary artery disease and chronic kidney disease across diabetic retinopathy stages.^9 10^

Traditionally, the assessment of diabetic retinopathy has been largely qualitative, relying on clinical examination to identify the presence and severity of characteristic lesions such as microaneurysms, haemorrhages and neovascularisation. However, advances in retinal imaging technologies have enabled a shift from purely qualitative assessments to quantitative retinovascular indices. Microvascular parameters such as fractal dimension, vessel density, width and tortuosity have been associated with the development and progression of diabetic retinopathy.^11^,^13^ Additionally, retinal neurodegeneration, particularly of the retinal nerve fibre layer (RNFL) and ganglion cell/inner plexiform layer (GC-IPL), has been associated with the development of microvascular diabetic retinopathy among individuals with diabetes.^12 14^ Beyond their relevance to diabetic retinopathy, quantitative retinal markers have also been correlated with systemic complications such as stroke and cardiovascular disease in individuals with diabetes.^15 16^

Given that PDR has a strong association with mortality, quantitative markers derived from retinal imaging of individuals with PDR could serve as prognostic indicators for mortality, highlighting those individuals most in need of close surveillance. Here, we investigated quantitative retinal morphological indices as prognostic markers for mortality in an urban ethnically diverse population with PDR.

## Methods

### Participants

This analysis uses data from the AlzEye cohort, a large retrospective cohort study of patients aged 40 years and over who have attended Moorfields Eye Hospital (MEH) in London, UK.^17^ Only individuals who had been newly diagnosed with PDR, as defined using the International Classification of Diabetic Retinopathy severity scale—neovascularisation and/or vitreous or preretinal haemorrhage—were included.^18^ A diagnosis was made using history, clinical examination and multimodal imaging interpreted by an ophthalmologist specialising in retina. Mortality data were derived from the MEH database, which is updated on a biweekly basis using reports extracted from the NHS National Spine and is completed on an individual basis by the MEH data quality team. We examined available patient data from 1 January 2013 to 1 April 2018. The primary objective was to assess the association between quantitative retinal features and time to death in individuals with PDR.

### Variables

All retinal images were acquired using Topcon devices (Topcon Corporation, Tokyo, Japan). Images from both eyes, where available, were used. From the patients with a diagnosis of PDR, we excluded patients who did not have imaging available within 3 months of diagnosis. For image selection, we chose the earliest imaging following diagnosis, to reduce the potential bias of patients receiving ophthalmic treatment for PDR (eg, intravitreal injections or panretinal photocoagulation). Retinal morphological features derived from macula-centred 45° colour fundus photography (CFP) and optical coherence tomography (OCT), acquired using the 3D OCT 2000 (Topcon, Tokyo, Japan). OCT images covered a 6.0 mm×6.0 mm area and had 128 horizontal B scans and 512 A scans per B scan; there was no B scan averaging. Retinal microvascular morphometric characteristics were extracted from CFP images using the deep learning pipeline AutoMorph.^19^ AutoMorph uses deep learning models for image quality grading and anatomical segmentation to produce the final output of measured vascular morphology features. We used CFP images with good or usable quality. More information on how image quality is categorised can be found in AutoMorph’s description.^19^ We also excluded OCT images with low quality and 10% of the most extreme values in certain imaging parameters such as the motion vector and boundary strength parameters commensurate with previous work in the UK Biobank cohort.^20 21^

We examined retinal vessel fractal dimension, vessel density, vessel tortuosity density, vessel average width and the central retinal arteriolar equivalent (CRAE)/central retinal venular equivalent (CRVE), which was calculated using the Knudtson-Parr-Hubbard formula. For the retinal sublayers, we examined the RNFL and GC-IPL. These retinal features were selected based on evidence in the literature demonstrating significant quantitative alterations of these features among individuals with diabetes mellitus and diabetic retinopathy.^11^,^13^ Retinal sublayers were defined according to the International Nomenclature for OCT panel,^22^ and thickness was estimated using the Topcon Advanced Boundary Segmentation Tool V.1.6.2.6.^23^ The parafoveal Early Treatment Diabetic Retinopathy Study grid regions were examined. All retinal feature values were standardised by centring at the mean and scaling by the SD before statistical analysis.

Secondary exposure variables were age, sex, socioeconomic deprivation, self-described ethnicity^24^ and hypertension (ICD-10: I10). Socioeconomic deprivation was estimated using the Index of Multiple Deprivation (IMD) from the 2015 IMD rank. The IMD is a composite score linked to postcodes that cover income, employment, education, health, barriers to housing and services, crime and living environment across England.^25^ The IMD rank is commonly reported by deciles, where decile 1 represents the most deprived 10% of the population.

### Statistical analyses

Continuous variables were compared between groups using the Wilcoxon-Mann-Whitney test and categorical variables through the χ^2^ test. Time to death was defined as the interval between the date of imaging and the date of death (all-cause mortality) with censoring at 1 April 2018 (end of study). Kaplan-Meier curves were plotted to illustrate survival probabilities stratified by quartiles of retinal feature values with comparison between groups using the log-rank test. HRs with 95% CIs were estimated using unadjusted and adjusted Cox-proportional hazards models with random effects for each retinal feature. Models were adjusted for age, sex, socioeconomic deprivation, image quality and comorbid hypertension. Data were analysed using the R V.4.3.2. The assumption of proportional hazards was tested using Schoenfeld residual tests.^26^

### Patient and public involvement

Patients and members of the public were involved in the conception and design of the AlzEye study through the National Institute for Health Research Biomedical Research Centre (BRC) at MEH and UCL Institute of Ophthalmology. Specifically, patients and the public were surveyed for their opinions of linking ophthalmic and systemic disease data. Patients and public members of the AlzEye working group will be invited to contribute to the dissemination of the study results.

## Results

**Figure 1:**
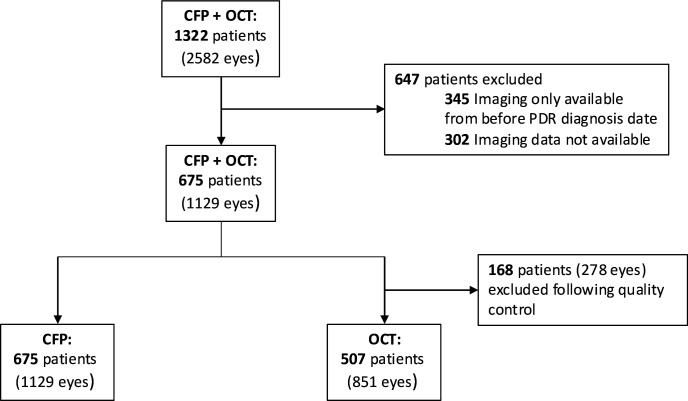
Flowchart of the patients included in the colour fundus photography and optical coherence tomography (OCT) cohorts. CFP, colour fundus photography; PDR, proliferative diabetic retinopathy.

During the study period, 1322 patients had a diagnosis of PDR and available imaging within 3 months of diagnosis in the AlzEye cohort. From the 1322 patients with PDR, 675 patients (1129 eyes) had macula centred CFP images that met our inclusion criteria. Of these 675 patients, 507 patients (851 eyes) also had OCT images of sufficient quality, respectively (figure 1).

**Figure 2:**
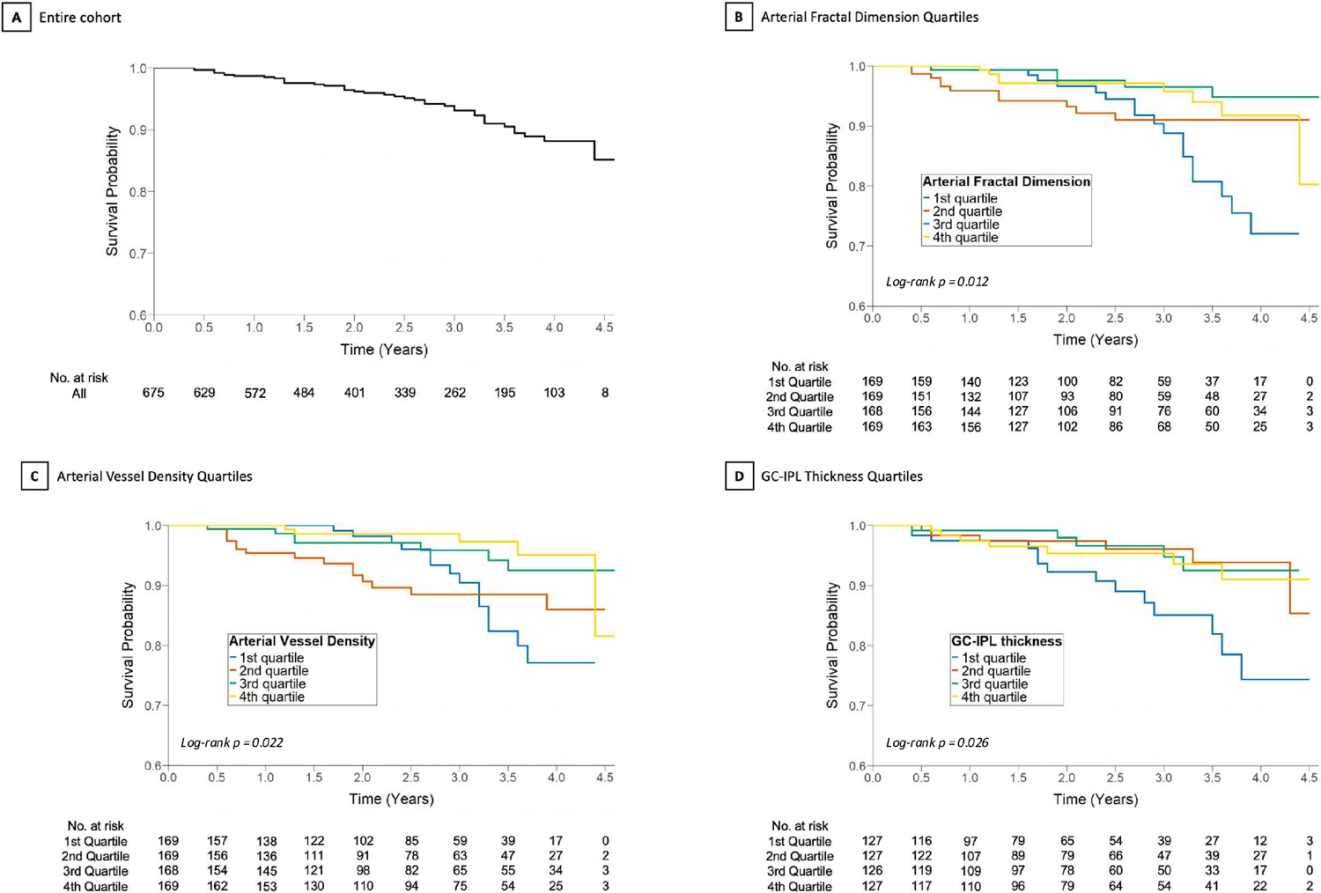
Kaplan-Meier cumulative survival estimates for (A) All patients in the colour fundus photography cohort, and those stratified by (B) quartiles of arterial fractal dimension, (**C**) quartiles of arterial vessel density. (**D**) All patients in the optical coherence tomography cohort stratified by quartiles of GC-IPL thickness quartiles (1st quartile: <25%, 2nd quartile: 25%–50%, 3rd quartile: 50%–75%, 4th quartile: >75%). Survival probability was plotted over the study period, with mortality as the primary event. GC-IPL, ganglion cell–inner plexiform layer.

In the CFP cohort, the mean (SD) age of included patients was 57.3 (±10.3) years (table 1), the majority of the cohort was male (60.7%) and individuals self-described as Asian were the largest ethnic group (37.6%). Hypertension was also largely prevalent in the cohort, with 483 (71.6%) patients having comorbid hypertension. During the study period, 6.1% (n=41) died. Based on Kaplan-Meier estimates, the 5-year survival probability was 85% (figure 2A). Baseline retinal morphology values are provided in online supplemental Table 1. To assess potential bias due to image availability, we examined baseline characteristics and the mortality rate in individuals excluded from the analysis due to unavailable imaging. The mean (SD) age in this cohort was 60.2 (±10.3), hypertension was present in 249 (82.5%) patients and the mortality risk in this group was 9.9% (n=30) (online supplemental Table 2).

**Table 1:** Summary patient characteristics of the colour fundus photography and optical coherence tomography cohorts

Characteristic	Colour fundus photography (n=675)	Optical coherence tomography (n=507)
Age, mean±SD (years)	57.3 (±10.3)	56.6 (±10.2)
Male sex, n (%)	410 (60.7)	318 (62.7)
Ethnicity, n (%)		
Asian Black White Other/mixed/unknown	254 (37.6) 84 (12.4) 158 (23.4) 179 (26.5)	189 (37.3) 62 (12.2) 115 (22.7) 141 (27.8)
Socioeconomic deprivation, mean±SD (IMD decile)	4.3 (±2.4)	4.3 (±2.4)
Hypertension, n (%)	483 (71.6)	353 (69.6)

IMD, Index of Multiple Deprivation.

On unadjusted analysis, decreases in arterial and venous fractal dimensions (HR: 1.54, 95% CI: 1.22 to 1.92 and HR: 1.37, 95% CI: 1.10 to 1.72), arterial and venous vessel density (HR: 1.67, 95% CI: 1.23 to 2.22) and HR: 1.49, 95% CI: 1.12 to 2.00), CRAE and CRVE (HR: 1.49, 95% CI: 1.16 to 1.92 and HR: 1.43, 95% CI: 1.05 to 1.92) and arterial average width (HR: 1.35, 95% CI: 1.02 to 1.79) were associated with an increased risk of death (table 2). There was no significant association between the risk of death and venous average width or arterial and venous tortuosity density.

**Table 2:** Association between time-to-death and retinal features from multimodal retinal imaging (HRs per SD decrease)

		Model 1*		Model 2^†^		Model 3‡	
		HR (95% CI)	P value	HR (95% CI)	P value	HR (95% CI)	P value
Colour fundus photography	Arterial fractal dimension	1.54 (1.22 to 1.92)	**<0.001**	1.54 (1.18 to 2.04)	**0.002**	1.45 (1.08 to 1.92)	**0.013**
	Venous fractal dimension	1.37 (1.10 to 1.72)	**0.005**	1.20 (0.90 to 1.61)	0.199	1.16 (0.86 to 1.59)	0.315
	Arterial vessel density	1.67 (1.23 to 2.22)	**<0.001**	1.59 (1.15 to 2.17)	**0.005**	1.47 (1.05 to 2.08)	**0.025**
	Venous vessel density	1.49 (1.12 to 2.00)	**0.007**	1.23 (0.88 to 1.75)	0.221	1.19 (0.84 to 1.69)	0.330
	Arterial average width, µm	1.35 (1.02 to 1.79)	**0.036**	1.35 (1.02 to 1.79)	**0.034**	1.28 (0.96 to 1.69)	0.086
	Venous average width, µm	1.10 (0.79 to 1.52)	0.583	1.05 (0.79 to 1.41)	0.709	1.05 (0.79 to 1.41)	0.728
	Arterial tortuosity density	1.12 (0.88 to 1.45)	0.353	1.06 (0.81 to 1.39)	0.652	1.01 (0.78 to 1.33)	0.911
	Venous tortuosity density	1.06 (0.79 to 1.41)	0.693	1.08 (0.87 to 1.33)	0.481	1.10 (0.88 to 1.37)	0.381
	CRAE Knudtson, µm	1.49 (1.16 to 1.92)	**0.002**	1.39 (1.05 to 1.82)	**0.018**	1.30 (0.99 to 1.72)	0.059
	CRVE Knudtson, µm	1.43 (1.05 to 1.92)	**0.020**	1.16 (0.85 to 1.59)	0.328	1.16 (0.85 to 1.59)	0.354
Optical coherence tomography	RNFL, µm	1.11 (0.76 to 1.61)	0.592	1.12 (0.78 to 1.61)	0.526	1.19 (0.81 to 1.59)	0.477
	GC-IPL, µm	1.79 (1.15 to 2.78)	**0.046**	1.61 (1.03 to 2.50)	**0.036**	1.56 (1.03 to 2.38)	**0.037**

Estimates are derived from unadjusted retinal nerve fibre layer.

Values in bold were considered statistically significant.

* Unadjusted Cox proportional hazards model.

† Adjusted for age, sex, socioeconomic deprivation and image quality.

‡ Additionally adjusted for hypertension.

CRAE, central retinal artery equivalent; CRVE, central retinal vein equivalent; GC-IPL, ganglion cell-inner plexiform layer; RNFL, retinal nerve fibre layer.

When adjusting for age, sex, socioeconomic deprivation, image quality and comorbid hypertension, the significant association was limited to arterial microvascular indices. On adjusted analysis, each 1-SD decrease in arterial fractal dimension was associated with a 45% higher hazard of death (HR: 1.45, 95% CI: 1.08 to 1.92). Each 1-SD decrease in arterial vessel density was associated with a 47% higher hazard of death (HR: 1.47, 95% CI: 1.05 to 2.08). There was no significant association between the risk of death and arterial average width (HR: 1.28, 95% CI: 0.96 to 1.69) and CRAE (HR: 1.30, 95% CI: 0.99 to 1.72).

On unadjusted and adjusted analysis of the retinal sublayers, a decrease in GC-IPL thickness was associated with an increased risk of death. When adjusting for demographic factors and comorbid hypertension, lower GC-IPL (per 1-SD decrease) was associated with an increased risk of death (HR: 1.56, 95% CI: 1.03 to 2.38, table 2).

To explore potential effect modification by sex and ethnic group, we conducted stratified Cox proportional hazards analyses. Among male participants, each 1-SD decrease in arterial fractal dimension, arterial vessel density and CRAE was significantly associated with increased mortality risk (HR: 1.75, 95% CI: 1.32 to 2.33; HR: 1.92, 95% CI: 1.35 to 2.70; HR: 1.45, 95% CI: 1.01 to 2.04, respectively). These associations were not statistically significant among female participants, as shown in online supplemental Table 3.

When stratified by ethnic group, each 1-SD decrease in arterial fractal dimension was significantly associated with higher mortality risk in individuals identifying as White (HR: 1.92, 95% CI: 1.14 to 3.23) and other/mixed/unknown individuals (HR: 1.67, 95% CI: 1.16 to 2.38), but not in those identifying as black or Asian (online supplemental Table 4). A similar pattern was observed for venous fractal dimension, with significant associations seen in white and other/mixed/unknown individuals (HR: 2.44, 95% CI: 1.52 to 4.00; HR: 1.75, 95% CI: 1.14 to 2.70). Lower arterial vessel density was also associated with increased mortality in the other/mixed/unknown group (HR: 1.96, 95% CI: 1.15 to 3.45), while lower venous vessel density was associated with increased mortality in white individuals (HR: 3.70, 95% CI: 1.69 to 7.69). Among all ethnic groups, only individuals identifying as black had a decreased mortality risk associated with lower retinal feature measurements. Specifically, each 1-SD decrease in venous fractal dimension, venous vessel density and CRVE was significantly associated with reduced mortality risk (HR: 0.43, 95% CI: 0.24 to 0.80; HR: 0.56, 95% CI: 0.35 to 0.90; HR: 0.60, 95% CI: 0.37 to 0.98, respectively).

## Discussion

This study provides new insights into the association between retinal features and mortality among individuals with diabetic retinopathy. We found that, in individuals with PDR, quantitative microvasculature alterations in arterial vessel parameters had a stronger association with mortality compared with venous vessel parameters. The retinal features significantly associated with mortality included arterial fractal dimension, arterial vessel density, arterial average width, CRAE and GC-IPL thickness after adjustment for multiple demographic factors which included age, sex, socioeconomic deprivation and image quality. The results from this study suggest that quantitative retinal markers could present a novel approach for identifying individuals with PDR who are most at risk of life-threatening sequelae.

Our study has several limitations. First, we had a high number of individuals with PDR who were excluded from the study. The majority were excluded because the available imaging was before PDR diagnosis. Patients were also excluded due to not having imaging available for analysis. Second, we had a low number of individuals with PDR included in our study. However, the AlzEye cohort represents a large patient population and this could be a general limitation of studying individuals with PDR. Third, we only included the first retinal images following PDR diagnosis which reduces the potential bias of patients receiving ophthalmic treatment for PDR; however, the treatments received by patients before PDR diagnosis are unknown and can be variable. Fourth, there were confounders that could not be adjusted for due to a lack of data, most notably medication history, cardiovascular risk factors, renal function and refractive error. Fifth, the cause of death of the patient cohort is unknown, although it is most likely a combination of stroke-related and cardiovascular mortality given its prevalence in patients with diabetic retinopathy.^9 10^

Arterial fractal dimension and arterial vessel density had the strongest association from the microvascular parameters as a significant relationship was seen even after adjusting for comorbid hypertension. A healthy individual’s retinal vascular network has been shown to have higher complexity or fractal dimension and vessel density compared with individuals with pathological processes, including PDR.^27^ Our finding of a decrease in fractal dimension and vessel density being associated with an increased risk of death is supported by the broader literature linking these retinal microvascular features to systemic health outcomes. In the general population, decreased fractal dimension and vessel density have been associated with an increased risk for stroke.^28^ Reduced retinal fractal dimension and reduced retinal vessel density have also been independently associated with an increased risk for stroke mortality, cardiovascular mortality and all-cause mortality.^29^,^31^ These findings suggest that less complex and less dense retinal vascular networks are reflective of worse systemic vascular health, which results in an increased mortality risk in individuals with PDR.

Quantitative microvascular decreases in fractal dimension and vessel density are reflective of arteriovenous differentiation in response to hypoxic signals from ischaemic retinal microcapillaries, which has been observed in embryological retinal vasculature.^32^ Neovascularisation produces polarised fractal canopies that have a smaller fractal dimension than normal retinal vascular networks.^32^ Greater ischaemic insults have also been previously suggested to cause larger decreases in vessel density.^33^ This suggests that retinal ischaemic changes may serve as a surrogate marker of increased mortality risk, reflecting broader systemic vascular dysfunction. Future research investigating more granular vascular changes of the posterior pole, such as with OCT angiography (OCTA), would help further characterise the relationship with mortality and non-ophthalmic microvascular sequelae.

Decreases in arterial average width and CRAE significantly increased risk of death following adjustment for demographic factors but were insignificant following additional adjustment for comorbid hypertension. From adaptive optics retinal imaging, individuals with PDR have been shown to have significantly higher vessel wall thickness and narrower lumen diameter compared with the other stages of diabetic retinopathy.^34^ Retinal vessel width can be influenced by several cardiovascular risk factors, including mean arterial blood pressure, duration of diabetes, glycaemic control, BMI and cholesterol levels,^35^ which could be potential confounders. Arterial average width and CRAE had statistical significance when adjusting for demographic factors, suggesting that these vessel parameters can be utilised as valid biomarkers for mortality risk stratification in diabetic retinopathy. Increased vessel wall thickness reduces retinal blood flow and contributes to retinal ischaemia,^34^ further supporting its potential as a prognostic marker for mortality among patients with diabetic retinopathy.

Surprisingly, we found that vessel tortuosity was not associated with mortality in individuals with PDR. Despite increased vessel tortuosity being associated with an increase in cerebrovascular events and all-cause mortality in patients with diabetes and the general population,^15 31^ there was no significant association in patients with PDR. On OCTA, an increase in the stage of NPDR has been associated with an increase in vessel tortuosity compared with healthy controls.^36^ In contrast, individuals with PDR have been noted to have a decrease in vessel tortuosity compared with healthy controls.^36^ The transition between NPDR and PDR vessel tortuosity can provide an explanation for the lack of a significant association with mortality. The exact pathophysiological mechanism of the decrease in vessel tortuosity that is unique to individuals with PDR is not known and further investigation into the potential prognostic value of vessel tortuosity in NPDR is needed.

Decreases in retinal GC-IPL thickness were also significantly associated with an increased risk of mortality in the current study. In patients with PDR, the RNFL and GC-IPL thicknesses were both significantly decreased when compared with patients with NPDR.^37^ Both GC-IPL and RNFL thicknesses are significantly decreased in PDR; however, only GC-IPL thickness had an association with mortality. There are studies that have suggested that the association between retinal neural thickness and the stage of diabetic retinopathy is not linear. Patients with moderate to severe NPDR have been found to have increased retinal thickness when compared with patients with mild NPDR and patients with PDR.^38^ Thickening in the foveal area is mainly attributed to thickening of the GC-IPL.^38^ GC-IPL thickness has been shown to decrease when progressing from moderate to severe NPDR to PDR, which is likely due to inner retinal atrophy from retinal ischaemia.^39^ The reduction in GC-IPL thickness reflects an increase in disease severity, and it could be a sensitive prognostic indicator for associated mortality.

Stratified analyses revealed that decreased arterial fractal dimension, arterial vessel density and CRAE were significantly associated with increased mortality risk among male participants, while these associations were not statistically significant in female participants. This sex-specific association is consistent with previous studies suggesting that anatomical and hormonal differences may impact the retinal microvasculature and influence its prognostic value. For example, in a stroke cohort, reduced fractal dimension was significantly associated with mortality in men but not in women,^29^ highlighting potential sex-based differences in microvascular vulnerability and its association with systemic vascular health.

When stratified by ethnic group, significant associations between lower arterial and venous fractal dimension and increased mortality risk were observed in individuals identifying as white and those in the other/mixed/unknown group, but not among individuals identifying as Black or Asian. This finding may reflect known variations in retinal vascular geometry across ethnicities, including vessel fractal dimension, calibre and tortuosity.^40 41^ Ethnic differences in cardiovascular risk profiles, systemic vascular health and microvascular remodelling may also contribute to the observed heterogeneity. Notably, only individuals identifying as black demonstrated decreased mortality risk associated with lower retinal feature measurements—including venous fractal dimension, venous vessel density and CRVE—suggesting distinct microvascular and/or systemic pathways influencing prognosis in this group.

Patients with PDR exhibit the highest prevalence of diabetic macular ischaemia (DMI) across the diabetic retinopathy stages.^42^ DMI on OCTA images has previously demonstrated prognostic value for diabetic retinopathy progression, diabetic macular oedema progression and visual acuity deterioration.^43^ In this study, we identified significant associations between quantitative retinal markers of retinal ischaemia—derived from CFP and OCT imaging—and mortality in patients with PDR. These findings suggest that retinal ischaemia may reflect broader systemic vascular pathology, warranting further investigation into its potential as a biomarker for long-term outcomes in patients with diabetic retinopathy.

## Conclusions

We found that several quantitative retinal markers have a significant association with mortality among individuals with PDR. The retinal features with a significant association included arterial fractal dimension, arterial vessel density, arterial average width, CRAE and GC-IPL thickness after adjustment for multiple demographic factors. Following adjustment for hypertension, statistical significance was seen in arterial fractal dimension, arterial vessel density and GC-IPL thickness. Importantly, stratified analyses revealed that these associations were more pronounced in male participants and varied by ethnic group, highlighting possible sex-specific and ethnicity-specific microvascular dynamics. These findings suggest that quantitative retinal markers of retinal ischaemia, derived from low-cost and widely available imaging modalities, could have prognostic value in mortality risk stratification of individuals with PDR. Further investigation into the association between quantitative retinal markers and diabetic retinopathy is needed for an improved understanding of the pathophysiological processes involved and their association with mortality. Longitudinal studies which examine microvascular indices and the risk of mortality following disease progression and treatment are warranted to better establish the association between quantitative retinal markers and mortality among individuals with PDR. Advanced retinal imaging modalities such as OCTA and adaptive optics retinal imaging can provide further information on the association of retinal features with mortality through depth-resolved imaging of retinal structure, vasculature and blood flow.

## Supplementary material

10.1136/bmjopen-2025-105231 online supplemental file 1

## Data Availability

Data are available on reasonable request. Data may be obtained from a third party and are not publicly available. No data are available.
