# Supplementary material for: Quantitative retinal morphology and mortality in individuals with proliferative diabetic retinopathy: a retrospective cohort study in a large real-world population
Source: BMJ Open. 2025 Sep 9;15(9):e105231. doi: 10.1136/bmjopen-2025-105231 (PMC12421592; doi:10.1136/bmjopen-2025-105231)
Supplement: online supplemental file 1 [file bmjopen-15-9-s001.docx]

|  | Mean (SD) |
| --- | --- |
| **Colour fundus photography** | Total  (n=1129 eyes) |
| Arterial fractal dimension | 1.22 (0.07) |
| Venous fractal dimension | 1.28 (0.07) |
| Arterial vessel density | 0.022 (0.007) |
| Venous vessel density | 0.031 (0.009) |
| Arterial average width, µm | 65.5 (9.2) |
| Venous average width, µm | 76.4 (10.5) |
| Arterial tortuosity density | 0.744 (0.067 |
| Venous tortuosity density | 0.764 (0.047) |
| CRAE Knudtson, µm | 145.4 (29.4) |
| CRVE Knudtson, µm | 251.1 (44.9) |
| **Optical coherence tomography** | Total  (n=851 eyes) |
| RNFL, µm | 31.6 (18.0) |
| GC-IPL, µm | 82.8 (18.0) |

Supplementary Table 1 : Summary image characteristics from the colour fundus photography and optical coherence tomography cohorts. CRAE: central retinal artery equivalent, CRVE: central retinal vein equivalent, GC-IPL: ganglion cell-inner plexiform layer, RNFL: retinal nerve fibre layer.

| **Characteristic** | Total  (n=302) |
| --- | --- |
| Age, mean +/- SD (years) | 60.2 (±10.3) |
| Male sex, n (%) | 177 (58.6) |
| Ethnicity, n (%)    Asian  Black  White  Other/Mixed/Unknown | 106 (35.1)  53 (17.5)  79 (26.2)  64 (21.2) |
| Socioeconomic deprivation, mean +/- SD (IMD decile) | 4.2 (±2.3) |
| Hypertension, n (%) | 249 (82.5) |
| Died during the study period, n (%) | 30 (9.9) |

Supplementary Table 2 : Baseline characteristics of the individuals who were excluded due to lack of available imaging. IMD: Index of multiple deprivation, SD: standard deviation.

|  | | **Male** | | **Female** | |
| --- | --- | --- | --- | --- | --- |
|  |  | **HR (95% CI)** | **p-value** | **HR (95% CI)** | **p-value** |
| **Colour fundus photography** | Arterial fractal dimension | 1.75 (1.32–2.33) | **<0.001** | 1.01 (0.52–1.92) | 0.983 |
|  | Venous fractal dimension | 1.23 (0.85–1.79) | 0.261 | 1.12 (0.71–1.79) | 0.616 |
|  | Arterial vessel density | 1.92 (1.35–2.70) | **<0.001** | 0.98 (0.55–1.75) | 0.942 |
|  | Venous vessel density | 1.25 (0.82–1.89) | 0.306 | 1.19 (0.70–2.04) | 0.508 |
|  | Arterial average width | 1.33 (0.90–1.96) | 0.152 | 1.18 (0.82–1.69) | 0.378 |
|  | CRAE Knudtson | 1.45 (1.01–2.04) | **0.042** | 1.12 (0.77–1.64) | 0.547 |
|  | CRVE Knudtson | 1.09 (0.71–1.67) | 0.689 | 1.39 (0.91–2.13) | 0.130 |
| **Optical coherence tomography** | GC-IPL | 1.64 (0.97–2.78) | 0.066 | 1.56 (0.79–3.13) | 0.201 |

Supplementary Table 3 : Association between time-to-death and retinal features from multimodal retinal imaging stratified by sex (HRs per standard deviation decrease). Estimates are derived from a Cox proportional hazards model adjusted for age, socioeconomic deprivation, image quality and hypertension. CRAE: central retinal artery equivalent, CRVE: central retinal vein equivalent, GC-IPL: ganglion cell-inner plexiform layer, RNFL: retinal nerve fibre layer.

|  |  | **Ethnic group** | **HR (95% CI)** | **p-value** |
| --- | --- | --- | --- | --- |
| **Colour fundus photography** | Arterial fractal dimension | White | 1.92 (1.14–3.23) | **0.014** |
|  |  | Black | 0.93 (0.48–1.82) | 0.838 |
|  |  | Asian | 1.19 (0.74–1.92) | 0.473 |
|  |  | Other/Mixed/Unknown | 1.67 (1.16–2.38) | **0.006** |
|  | Venous fractal dimension | White | 2.44 (1.52–4.00) | **<0.001** |
|  |  | Black | 0.43 (0.24–0.80) | **0.008** |
|  |  | Asian | 0.98 (0.63–1.54) | 0.948 |
|  |  | Other/Mixed/Unknown | 1.75 (1.14–2.70) | **0.011** |
|  | Arterial vessel density | White | 2.38 (0.99–5.88) | 0.052 |
|  |  | Black | 1.00 (0.53–1.88) | 1.000 |
|  |  | Asian | 1.19 (0.73–1.92) | 0.487 |
|  |  | Other/Mixed/Unknown | 1.96 (1.15–3.45) | **0.014** |
|  | Venous vessel density | White | 3.70 (1.69–7.69) | **<0.001** |
|  |  | Black | 0.56 (0.35–0.90) | **0.016** |
|  |  | Asian | 1.05 (0.67–1.64) | 0.828 |
|  |  | Other/Mixed/Unknown | 1.79 (0.93–3.33) | 0.084 |
|  | Arterial average width | White | 1.03 (0.41–2.56) | 0.956 |
|  |  | Black | 1.12 (0.68–1.85) | 0.660 |
|  |  | Asian | 1.28 (0.79–2.08) | 0.310 |
|  |  | Other/Mixed/Unknown | 1.43 (0.56–3.57) | 0.439 |
|  | CRAE Knudtson | White | 1.56 (0.75–3.23) | 0.232 |
|  |  | Black | 1.11 (0.58–2.08) | 0.761 |
|  |  | Asian | 1.32 (0.93–1.89) | 0.118 |
|  |  | Other/Mixed/Unknown | 1.09 (0.45–2.63) | 0.858 |
|  | CRVE Knudtson | White | 1.85 (0.64–5.26) | 0.259 |
|  |  | Black | 0.60 (0.37–0.98) | **0.040** |
|  |  | Asian | 1.30 (0.32–5.26) | 0.222 |
|  |  | Other/Mixed/Unknown | 1.04 (0.44–2.44) | 0.717 |
| **Optical coherence tomography** | GC-IPL | White | 1.49 (0.57–4.00) | 0.931 |
|  |  | Black | 1.72 (0.74–4.00) | 0.412 |
|  |  | Asian | 0.65 (0.23–1.89) | 0.208 |
|  |  | Other/Mixed/Unknown | 1.92 (1.14–3.23) | 0.435 |

Supplementary Table 4 : Association between time-to-death and retinal features from multimodal retinal imaging stratified by ethnic group (HRs per standard deviation decrease). Estimates are derived from a Cox proportional hazards model adjusted for age, sex, socioeconomic deprivation, image quality and hypertension. CRAE: central retinal artery equivalent, CRVE: central retinal vein equivalent, GC-IPL: ganglion cell-inner plexiform layer, RNFL: retinal nerve fibre layer.
