# Supplementary material for: Quantitative retinal morphology and mortality in individuals with proliferative diabetic retinopathy: a retrospective cohort study in a large real-world population
Source: BMJ Open. 2025 Sep 9;15(9):e105231. doi: 10.1136/bmjopen-2025-105231 (PMC12421592; doi:10.1136/bmjopen-2025-105231)
Supplement: Supplementary file 2 [file bmjopen-2025-105231.reviewer_comments.pdf]

PEER REVIEW HISTORY

BMJ Open publishes all reviews undertaken for accepted manuscripts. Reviewers are asked to complete a checklist review form and are provided with free text boxes to elaborate on their assessment. These free text comments are reproduced below.

ARTICLE DETAILS

Title (Provisional)

Quantitative retinal morphology and mortality in individuals with proliferative diabetic retinopathy: a retrospective cohort study in a large real-world population

Authors

Khan, Abdullah Zafar; Ribeiro Reis, Ana Paula; Olvera-Barrios, Abraham; Zhou, Yukun; Williamson, Dominic J; Struyyen, Robbert R; Khalid, Hagar; Egan, Catherine; Denniston, Alastair K; Keane, Pearse; Wagner, Siegfried Karl

---

VERSION 1 - REVIEW

**Reviewer**	**1**
**Name**	**Owusu-Afriyie, Bismark**
**Affiliation**	**University of Houston College of Optometry**
**Date**	**21-Jun-2025**
**COI**	**None**

Thank you for the opportunity to review this important and timely manuscript, which explores the prognostic value of retinal imaging in patients with PDR. The study addresses a critical area in diabetic eye care by investigating the potential link between retinal biomarkers and mortality risk. Overall, the manuscript offers valuable insights but would benefit from clarification in a few areas to strengthen its scientific rigor and clinical relevance.

Abstract

1. Results Lines 40-42: Please revise “57.3 years [10.3]” to “57.3 [10.3] years” and revisit the calculation of the percentage. Kindly revise when and how you use “[ ]” and “)” in this section.
2. Results Line 49: This is the first mention of CRAE in the manuscript. It may be helpful to provide the full meaning if word count does not limit the authors.

Introduction

3. Lines 13-17: Please provide the year for the estimated prevalence of diabetic retinopathy and vision-threatening diabetic retinopathy in the US.
4. Since the study was conducted in the UK, it would be helpful for readers if the authors included information on the prevalence of diabetes and diabetic retinopathy within the UK population in the introduction.

Methods

5. Page 9, line 5: Kindly provide detailed information on the scan protocol used for acquiring the OCT images, including parameters such as scan type (e.g., raster, radial), scan area, resolution, number of B-scans, and any averaging or enhancement techniques applied.
6. The authors are kindly requested to indicate whether OCTA images were available for the patients. If so, it would be valuable to compare the vascular indices derived from OCTA with those obtained from CFP. Additionally, please clarify whether the use of OCTA data yielded similar levels of statistical significance and consistent directional trends in the results (if OCTA data is available).
7. The authors should please indicate whether corrections were made for axial length, corneal curvature, and refractive errors in their imaging data analyses, as these ocular parameters can influence vascular measurements.
8. Page 11, line 5: Please change "patient" to "patients." That is, "Patients and public members…."

Results

9. Percentages: Kindly revise the calculation of the percentages (including those of Table 1 and Supplementary Table 2), as some appear to be incorrect. For example, 37.8% should be used instead of 37.6%, and 71.6% instead of 71.3%, among others.
10. Table 1: Since the abbreviations CFP and OCT were not used within the Table itself, it may be unnecessary to define or explain them in the Table legend. You may consider removing these definitions.
11. Figure 1: In the title, please consider replacing the word "into" with "in"
12. Figure 1: Please change "365 measurements" to "365 eyes" for clarity, since the measurements can refer to either eyes or individuals.
13. Page 12, Lines 41-44: The authors stated that "….and arterial average width (HR 0.74, 95%CI 0.56-0.98) were associated with an increased risk of death (Supplementary

Table 2, Table 2). However, supplementary Table 2 presents the baseline characteristics of the excluded patients; it does not include any analysis of associations. Kindly check the table and clarify this statement.

14. Supplementary Table 1: Please clarify the meaning of the numbers in parentheses, as was done for Table 1. Indicating whether they represent percentages, standard deviations, or another measure will enhance clarity and consistency.
15. Page 12, Line 54: Please indicate that the association was significant. That is, "…and co-morbid hypertension, the significant association was limited to…"
16. The authors should kindly clarify if they observed racial and sex differences in the indices they measured.

Discussion

1. Page 14, lines 43: Please include the number for the supplementary table.
2. Page 15, line 34: Kindly put a period (full stop) after the citation as you have done for all the others. That is, "………and all-cause mortality [28-30]. These findings…"
3. Page 16, lines 46-54: Please provide citation and reference for the statement "In contrast, individuals with PDR have been noted to have a decrease in vessel tortuosity compared to healthy controls."
4. Page 16, lines 56: Please insert the phrase "in the current study" after the word "mortality" for clarity. That is, "…..mortality in the current study."

References

1. Kindly provide the full citation for reference number 2 to allow readers to locate the source easily.
2. The use of all capital letters (upper case) for the title of reference number 35 is inconsistent with the formatting of the other references. Please revise it to match the standard format used throughout the reference list, taking into consideration the guidelines of the journal.

**Reviewer**	**2**
**Name**	**Chung, Yoo-Ri**
**Affiliation**	**Ajou University School of Medicine**
**Date**	**23-Jun-2025**

**COI**	**None**

Authors in the manuscript titled “Retinal imaging as a prognostic factor for mortality in patients with proliferative diabetic retinopathy” have investigated the role of quantitative retinal imaging markers as potential prognostic factors for mortality in PDR patients.

This study has significant methodological problems that need to be considered.

Major comments:

Do the CFP cohort and the OCT cohort overlap or not? There is no explanation on this, which is a basic content.

Considering that the numbers of total patients, CFP cohort, and OCT cohort are 1322, 675, and 507 patients, I cannot understand where the number of ‘15% (n=41) died’ comes from. This is one of the most important number of the results. What is the denominator?

Similarly, what is the denominator for ‘the mortality risk in this group was 9.9% (n=30)’? Moreover, the authors cited the Supplementary Table 1 here, which does not include the aforementioned information.

The paragraph citing the supplementary Table 2 in Results section and the table itself do not correlate. The supplementary Table 2 presents only demographics of excluded patients.

The authors already mentioned that the cause of death was not investigated in the limitations. However, this is critical to this study investigating retinal vascular parameters, as diabetic patients are prone to not only cardiovascular diseases but infection (sepsis).

Dialysis and intravitreal injections of anti-VEGF agents can be significant confounding factor to investigate mortality in diabetic patients, which were not considered in HR adjustment.

Results on Kaplan-Meier analyses are only presented as figure 2, whereas they are absent in the results section. Moreover, the log-rank p-vales are lacking.

Minor comments:

In Supplementary Table 1, the number presented as OO (OO) should be explained. I suppose that these are means and standard deviations, but this should be stated.

In Supplementary Table 1, the number mentioned at the head of the table is that of the patients or eyes? There is no explanation.

VERSION 1 - AUTHOR RESPONSE

Reviewer 1

Comment	Author Response	Changes made
**Abstract**		
Results Lines 40-42: Please revise “57.3 years [10.3]” to	Thank you. We have revised the abstract result lines 40-42	Abstract<br>Results

"57.3 [10.3] years" and revisit the calculation of the percentage. Kindly revise when and how you use "[ ]" and ")" in this section.		"A total of 675 individuals (1129 eyes) with PDR were included, with a mean age of 57.3 years (SD 10.3); 410 (60.7%) were male."
Results Line 49: This is the first mention of CRAE in the manuscript. It may be helpful to provide the full meaning if word count does not limit the authors.	We agree that since this is the first mention, the full meaning would be helpful and we have included it in the abstract	Abstract<br>Results<br>"…central retinal arteriolar equivalent (CRAE) (HR: 0.72, 95% CI: 0.55–0.95), and ganglion cell–inner plexiform layer (GC-IPL) thickness"
**Introduction**		
Lines 13-17: Please provide the year for the estimated prevalence of diabetic retinopathy and vision-threatening diabetic retinopathy in the US.	We have added the year for the estimated prevalence of diabetic retinopathy and vision-threatening diabetic retinopathy in the US	Introduction<br>Para 1<br>"In the United States in 2021, it was estimated that 9.60 million people (26% of those with diabetes) had diabetic retinopathy and 1.84 million people (5% of those with diabetes) had vision-threatening diabetic retinopathy [4]"
Since the study was conducted in the UK, it would be helpful for readers if the authors included information on the prevalence of diabetes and diabetic retinopathy within the UK population in the introduction.	We appreciate the comment from the reviewer and have included information about the prevalence of diabetes in the UK. However, recent data on the prevalence of diabetic retinopathy in the UK remains limited, particularly within the last five years	Introduction<br>Para 1<br>"In the United Kingdom, as of 2024, more than 5.8 million people are estimated to be living with diabetes [3]"
**Methods**		
Page 9, line 5: Kindly provide detailed information on the scan protocol used for acquiring the OCT images, including parameters such as scan type (e.g., raster, radial), scan area, resolution, number of B-scans, and any averaging or enhancement techniques applied.	Thank you for highlighting this. We have added further details.	Methods<br>Variables<br>"OCT images covered a 6.0 mm × 6.0 mm^2 area and had 128 horizontal B scans and 512 A scans per B scan; there was no B scan averaging."
The authors are kindly requested to indicate whether OCTA images were	We thank the reviewer for this comment and agree that comparing the vascular	Discussion<br>Para 4

available for the patients. If so, it would be valuable to compare the vascular indices derived from OCTA with those obtained from CFP. Additionally, please clarify whether the use of OCTA data yielded similar levels of statistical significance and consistent directional trends in the results (if OCTA data is available).	indices derived from OCTA with CFP would provide valuable insights. Unfortunately, OCTA imaging was not available within the AlzEye cohort used in this study. We hope that the combination of CFP and OCT data adequately supports the analyses presented. We have added OCTA as an area for future research in the Discussion.	"Future research investigating more granular vascular changes of the posterior pole, such as with OCT angiography, would help further characterise the relationship with mortality and non-ophthalmic microvascular sequelae."
The authors should please indicate whether corrections were made for axial length, corneal curvature, and refractive errors in their imaging data analyses, as these ocular parameters can influence vascular measurements.	We agree with the Reviewer that these have implications for magnification and retinal sublayer thicknesses. Unfortunately we do not have this data available routinely and have therefore added this as among the potential sources of residual confounding.	Discussion<br>Para 2<br>"Fourth, there were confounders that could not be adjusted for due to a lack of data, most notably medication history, cardiovascular risk factors and refractive error."
Page 11, line 5: Please change "patient" to "patients." That is, "Patients and public members…."	We thank the reviewer for this comment and have corrected the spelling error	Methods<br>Patient and public involvement<br>"Patients and public members of the AlzEye working group will be invited to contribute to dissemination of the study results"
**Results**		
Percentages: Kindly revise the calculation of the percentages (including those of Table 1 and Supplementary Table 2), as some appear to be incorrect. For example, 37.8% should be used instead of 37.6%, and 71.6% instead of 71.3%, among others.	We thank the reviewer for this comment. We have corrected the numbers and percentages in Table 1. However, we did not make any amendments to Supplementary Table 2 as we did not find any inaccurate numbers or calculations	Table 1<br>Corrected numbers and percentage calculations
Table 1: Since the abbreviations CFP and OCT were not used within the Table itself, it may be unnecessary to define or explain them in the Table legend. You may consider removing these definitions.	Thank you. We have removed the definitions for the abbreviations	Table 1<br>Title<br>Removed definitions of abbreviations CFP and OCT

Figure 1: In the title, please consider replacing the word “into” with “in”	We have replaced the word “into” with “in” for the title of figure 1	Figure legends<br>Fig.1 title<br>“Flowchart of the patients included in the colour fundus photography and optical coherence tomography cohorts”
Figure 1: Please change “365 measurements” to “365 eyes” for clarity, since the measurements can refer to either eyes or individuals.	We thank the reviewer for this comment and have the changed “365 measurements” to “365 eyes” in figure 1	Figure 1<br>Changed “365 measurements” to “365 eyes”
Page 12, Lines 41-44: The authors stated that “….and arterial average width (HR 0.74, 95%CI 0.56-0.98) were associated with an increased risk of death (Supplementary Table 2, Table 2). However, supplementary Table 2 presents the baseline characteristics of the excluded patients; it does not include any analysis of associations. Kindly check the table and clarify this statement.	Thank you. We have corrected the mistake and removed the reference to Supplementary Table 2	Results<br>Para 3<br>“arterial average width (HR 0.74, 95% CI 0.56-0.98) were associated with an increased risk of death (Table 2).”
Supplementary Table 1: Please clarify the meaning of the numbers in parentheses, as was done for Table 1. Indicating whether they represent percentages, standard deviations, or another measure will enhance clarity and consistency.	We thank the reviewer for this comment. We have added a row and heading in Supplementary Table 1 that shows that the numbers represent the mean and standard deviation	Supplementary Table 1<br>Row 1<br>Added row 1 and “Mean (SD)” heading
Page 12, Line 54: Please indicate that the association was significant. That is, “…and co-morbid hypertension, the significant association was limited to…”	Thank you. We have indicated that the association was significant	Results<br>Para 4<br>“When adjusting for age, sex, socioeconomic deprivation, image quality and co-morbid hypertension, the significant association was limited to arterial microvascular indices.”

The authors should kindly clarify if they observed racial and sex differences in the indices they measured.	We thank the reviewer for this important point. We initially overlooked this and did not perform stratified analyses to explore the racial and sex differences in the indices we measured.<br><br>We have now performed stratified analyses based on the ethnic groups and sex and have found valuable insights. We have added Supplementary Table 3 and Supplementary Table 4 which include the results. We have also added the results to the Results section and we discuss the prognostic significance among different demographic subgroups in the Discussion section	Supplementary Tables<br>Added Supplementary Tables 3 and 4<br><br>Results<br>Para 6 and 7<br>Added the results from stratified analyses based on sex and ethnic group<br><br>Discussion<br>Para 9 and 10<br>Included discussion on the differential prognostic significance among the different sexes and ethnic groups
**Discussion**		
Page 14, lines 43: Please include the number for the supplementary table.	We have included the number for the supplementary Table	Discussion<br>Para 2<br>“However, the patient characteristics and the rate of death over the study period in the patients without available imaging has shown to be similar to the included cohort (Supplementary Table 2).”
Page 15, line 34: Kindly put a period (full stop) after the citation as you have done for all the others. That is, “………and all-cause mortality [28-30]. These findings…”	Thank you. We have added a period following the citation.	Discussion<br>Para 3<br>“…and all-cause mortality [29-31].”
Page 16, lines 46-54: Please provide citation and reference for the statement “In contrast, individuals with PDR have been noted to have a decrease in vessel tortuosity compared to healthy controls.”	Thank you. We have provided a citation and reference for the mentioned statement	Discussion<br>Para 6<br>“In contrast, individuals with PDR have been noted to have a decrease in vessel tortuosity compared to healthy controls [36].”

Page 16, lines 56: Please insert the phrase "in the current study" after the word "mortality" for clarity. That is, "…..mortality in the current study."	We have added "in the current study" to the mentioned sentence	Discussion<br>Para 7<br>"Decreases in retinal GC-IPL thickness were also significantly associated with an increased risk of mortality in the current study."
**References**		
Kindly provide the full citation for reference number 2 to allow readers to locate the source easily.	We have updated the introduction to include the latest information from 2024 and have added the full citation for reference number 2	Introduction<br>Para 1<br>"In 2024, 589 million adults across the world were estimated to have diabetes [2]."<br><br>References<br>Reference 2<br>"International Diabetes Federation (2024) Fact sheets: Diabetes around the world in 2024. Available from: (url)"
The use of all capital letters (upper case) for the title of reference number 35 is inconsistent with the formatting of the other references. Please revise it to match the standard format used throughout the reference list, taking into consideration the guidelines of the journal.	Thank you. We have made the reference consistent with the formatting of the other references	References<br>Reference 36<br>"Lee H, Lee M, Chung H, Kim HC (2018) Quantification of retinal vessel tortuosity in diabetic retinopathy using optical coherence tomography angiography. Retina 38(5): 976-985. 10.1097/iae.0000000000001618"

Reviewer 2

Comment	Author Response	Changes made
Do the CFP cohort and the OCT cohort overlap or not? There is no explanation on this, which is a basic content.	We thank the reviewer for this comment and appreciate that this could be made clearer.<br><br>We have sought to clarify this both in the text and the Figure.	Figure 1 updated.<br><br>Results:<br>Para 1<br>"From the 1322 patients with PDR, 675 patients (1129 eyes) had macula centred CFP images that met our inclusion criteria. Of these 675 patients, 507 patients (851 eyes) also had OCT images of sufficient quality respectively (Figure 1)."

Considering that the numbers of total patients, CFP cohort, and OCT cohort are 1322, 675, and 507 patients, I cannot understand where the number of '15% (n=41) died' comes from. This is one of the most important number of the results. What is the denominator?	Thank you for your comment on this important point. We have corrected the percentage calculation. The denominator is the number of patients in the CFP cohort and the calculation should have been: 41/675 = 6.1% and has now been corrected. We have also stated the survival probability estimate to be 85% based on the Kaplan-Meier curve in Figure 2	Results<br>Para 2<br>"During the study period, 6.1% (n=41) died. Based on the Kaplan-Meier estimates, the 5-year survival probability was 85% (Figure 2)."
Similarly, what is the denominator for 'the mortality risk in this group was 9.9% (n=30)'? Moreover, the authors cited the Supplementary Table 1 here, which does not include the aforementioned information.	We thank the reviewer for this comment. We have corrected the reference to Supplementary Table 2 which includes the baseline characteristics and mortality rate for the cohort of excluded patients. As indicated, the mortality proportion is 9.9%, calculated as 30 deaths among 302 patients (30/302)	Results<br>Para 2<br>"…mortality risk in this group was 9.9% (n=30) (Supplementary Table 2)."
The paragraph citing the supplementary Table 2 in Results section and the table itself do not correlate. The supplementary Table 2 presents only demographics of excluded patients.	Thank you. We have corrected the mistake and removed the reference to Supplementary Table 2	Results<br>Para 3<br>"arterial average width (HR 0.74, 95% CI 0.56-0.98) were associated with an increased risk of death (Table 2)."
The authors already mentioned that the cause of death was not investigated in the limitations. However, this is critical to this study investigating retinal vascular parameters, as diabetic patients are prone to not only cardiovascular diseases but infection (sepsis).	Thank you for this comment. We agree with the Reviewer that there are several potential causes of mortality in this comorbid patient group, from cardiovascular diseases to sepsis though the vast majority is due to CVD (https://www.ahajournals.org/doi/10.1161/JAHA.118.011295).<br><br>Nonetheless, we would highlight that the purpose of retinal imaging, in our view,	Discussion<br>Para 2<br><br>"Fifth, the cause of death of the patient cohort is unknown, although it is most likely a combination of stroke-related and cardiovascular mortality given its prevalence in patients with diabetic retinopathy [9, 10]."

	would not be to highlight the individual potential cause of death (whether it be CVD versus end stage renal failure vs infection vs cancer vs liver disease). It instead would provide a broad snapshot of medical morbidity burden through national screening programmes with referrals potentially going to diabetes services (which are increasingly multidisciplinary) as well as diabetic eye services.<br><br>The lack of cause of death data is a limitation of the current work and we have been explicit with this in the manuscript. We are also currently amending our study to obtain Civil Registrations cause of death data and have added a remark on this.	
Dialysis and intravitreal injections of anti-VEGF agents can be significant confounding factor to investigate mortality in diabetic patients, which were not considered in HR adjustment.	Thank you for this comment. This cohort relates to a newly referred group of treatment-naïve patients with proliferative diabetic retinopathy. They will therefore not have had any anti-VEGF agents.<br><br>We agree with the Reviewer that individuals with dialysis are likely to have different mortality patterns than those who are not on dialysis. While we agree with the Reviewer on this point, we would highlight that the objective of this work is not investigating the causal relationship between retinal morphology and mortality. Instead, we are investigating a prognostic use case - whether retinal imaging can identify those at high risk of mortality. While inclusion of	Discussion<br>Para 2<br><br>"Fourth, there were confounders that could not be adjusted for due to a lack of data, most notably medication history, cardiovascular risk factors, renal function and refractive error."

	dialysis use may improve the discrimination, this level of data is not routinely available in screening programmes. To highlight the Reviewer's point, we will include renal function as a key marker in the limitations section re: residual confounding.	
Results on Kaplan-Meier analyses are only presented as figure 2, whereas they are absent in the results section. Moreover, the log-rank p-vales are lacking.	We appreciate this comment from the reviewer. We have referenced figure 2 in the results section and have added the log-rank p-values to the figure.	Results<br>Para 2<br>"Based on Kaplan-Meier estimates, the 5-year survival probability was 85% (Figure 2)."<br><br>Figure 2<br>Added log-rank p-values
In Supplementary Table 1, the number presented as OO (OO) should be explained. I suppose that these are means and standard deviations, but this should be stated.	Thank you for this comment. We have added a row and heading for Supplementary Table 1	Supplementary Table 1<br>Row 1<br>Added row 1 and "Mean (SD)" heading
In Supplementary Table 1, the number mentioned at the head of the table is that of the patients or eyes? There is no explanation.	Thank you. We have included "eyes" in the subheadings of Supplementary Table 1	Supplementary Table 1<br>Row 2 and Row 13<br>"(n=1129 eyes)" and "(n=851 eyes)"

VERSION 2 - REVIEW

Reviewer 1

Name Owusu-Afriyie, Bismark

Affiliation University of Houston College of Optometry

Date 07-Aug-2025

COI

Methods: Please remove the square (that is 2) after the 6.0mm2 in the statement "OCT images covered a 6.0 mm × 6.0 mm2 area.....". You can change it to "6.0mm x 6.0mm area" OR "6.0 x 6.0 mm2 area"

Please revise the calculations in Table 1. 62/507 should be 12.2 and not 12.3

Likewise, 53/302 should be 17.5 instead of 17.6 in Supp Table 2.

VERSION 2 - AUTHOR RESPONSE

Reviewer 1

Comment	Author Response	Changes made
Methods: Please remove the square (that is 2) after the 6.0mm2 in the statement "OCT images covered a 6.0 mm × 6.0 mm2 area.....". You can change it to "6.0mm x 6.0mm area" OR "6.0 x 6.0 mm2 area"	Thank you, we have corrected this.	Square figure removed.
Please revise the calculations in Table 1. 62/507 should be 12.2 and not 12.3 Likewise, 53/302 should be 17.5 instead of 17.6 in Supp Table 2.	Thank you for identifying these errors. We have corrected them.	Values updated in Table 1 and Supplementary Table 2.
